# Submandibular Gland Basal Cell Adenoma

**DOI:** 10.22038/IJORL.2021.58329.3007

**Published:** 2022-03

**Authors:** Mohamad Najib Salleh, Abdul Azim Al-Abrar Ahmad Kailani, Nik Fatin Amirah Nik Min, Irfan Mohamad, Faezahtul Arbaeyah Hussain, Anani Aila Mat Zin

**Affiliations:** 1 *Department of Otorhinolaryngology-Head & Neck Surgery, School of Medical Sciences, Universiti Sains Malaysia Health Campus, 16150 Kota Bharu, Kelantan, Malaysia.*; 2 *Department of Pathology, School of Medical Sciences, Universiti Sains Malaysia Health Campus, 16150 Kota Bharu, Kelantan, Malaysia.*

**Keywords:** Biopsy, Cytology, Computed tomography, Salivary gland

## Abstract

**Introduction:**

Basal cell adenoma (BCA) is a rare benign epithelial tumour of the salivary gland majorly involving the parotid gland, and rarely the submandibular gland.

**Case Report:**

We describe a rare case of BCA of the submandibular gland diagnosed preoperatively using fine needle aspiration cytology in a 60-year-old woman presenting with painless submandibular swelling. The surgery went uneventfully, and the histopathological examination confirmed the diagnosis.

**Conclusions:**

BCA can be accurately diagnosed only through histological observations due to its resemblance to various benign and malignant salivary and non-salivary gland tumours, which are difficult to biopsy.

## Introduction

Tumours of the salivary gland represent approximately 3–4% of all neoplasms in the head and neck (1), around 80% of which arise in the parotid gland and rarely in the submandibular glands. Basal cell adenoma (BCA) is a benign salivary gland epithelial tumour, most commonly developing in the parotid and minor salivary glands, and it has distinct histological features from mixed tumours (1). 

## Case Report

**Fig 1:**
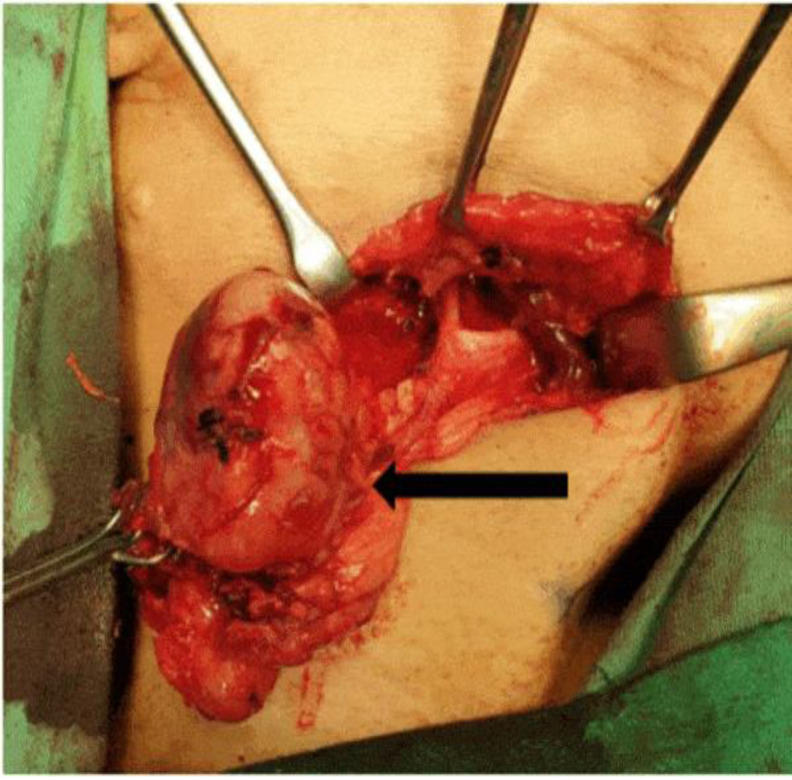
Right submandibular mass intra-operative (arrow) was consistent with benign lesion features of the submandibular gland

A 60-year-old female presented with slow-growing right submandibular swelling in the past year without any other associated symptoms, such as pain, difficulty in mouth opening, odynophagia, dysphagia, foreign body sensation, or hoarseness. She also denied loss of appetite, loss of weight, and prolonged fever. Upon physical examination, there was a right submandibular mass measuring 4 × 3 cm—firm in consistency, mobile, and ballotable (Figure 1). 

**Fig 2:**
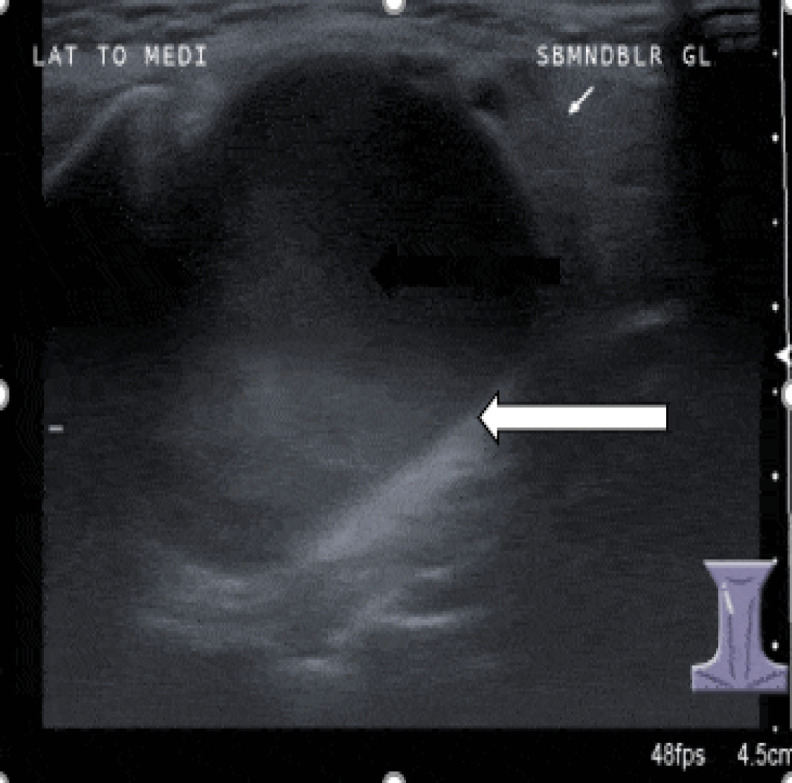
A well-defined heterogeneous solid lesion at right submandibular region (thick arrow). The lesion displaced the adjacent right submandibular gland (thin arrow)

There were no palpable lymph nodes at any level of the neck; examination of the ears, nose, and throat was also unremarkable. Ultrasonography of the neck revealed a well-defined solid lesion at the right submandibular region with heterogeneous echogenicity and measuring 3.0 cm (AP) × 3.5 cm (W) × 3.6 cm (CC); there was no calcification or cystic component within (Figure 2). 

 The patient was further investigated with ultrasound-guided Tru-Cut biopsy of the mass, as the fine needle aspiration for cytology (FNAC) was unsatisfactory. The biopsy showed nests of tumour cells with interlacing cords, clusters, and some trabecular patterns. The tumour cells were composed of basaloid cells with uniform round to oval hyperchromatic nuclei and scanty cytoplasm, suggestive of BCA. Subsequently, she underwent a right submandibulectomy. The surgery was uneventful, and the intraoperative findings were consistent with the features of a benign lesion.

The histopathological examination of the specimen showed a well-circumscribed encapsulated tumour composed of basal cell proliferation arranged in trabeculae, tubular, and solid patterns with peripheral palisading. The cells were uniform round-to-oval, with vesicular nuclei and scanty cytoplasm(Figure 3). The adjacent salivary gland did not show any signs of infiltration or invasion, and no evidence of malignancy was seen. Throughout our six-month follow-up after surgery, her wound healed well, and there were no clinical signs of recurrence.

**Fig 3 A:**
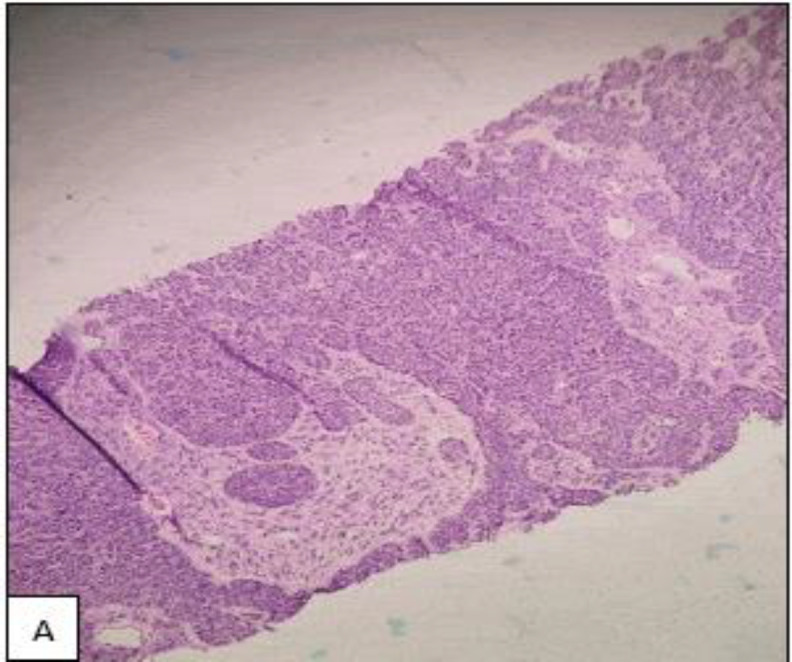
Hematoxylin and eosin (H&E) x 4 magnification of Tru-Cut biopsy proliferation of basal cells in trabecular and solid pattern

**Fig 3 B:**
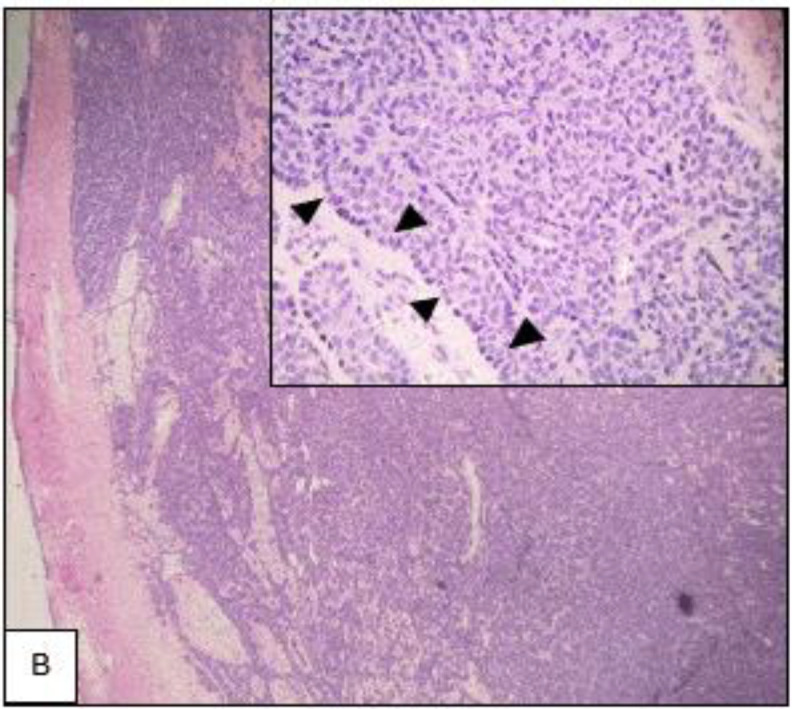
(H&E) x 40 magnification of excision of the tumour showing fairly circumscribed covered with fibrous capsule basal cell proliferation. The cells are round to oval, fairly uniform vesicular nuclei arranged in trabecular and solid pattern

**Fig 3 C:**
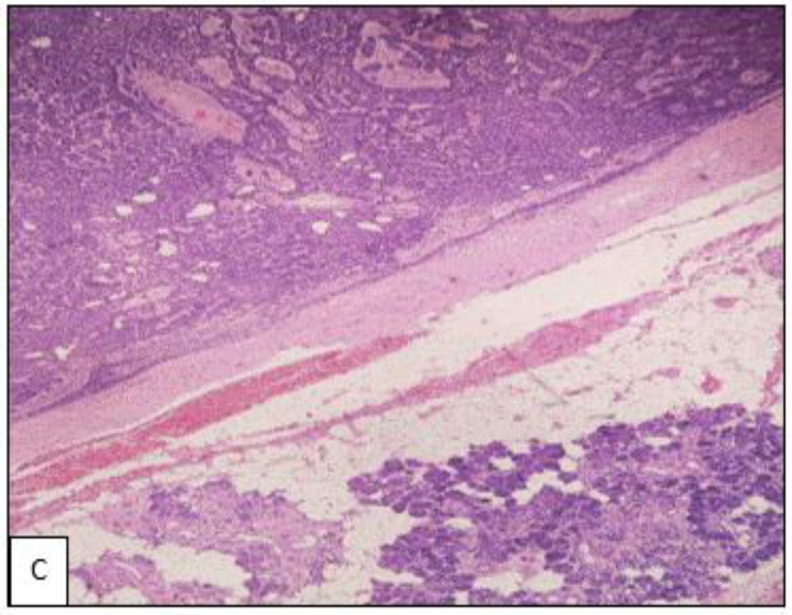
H&E x 100 magnification shows the tumour is well demarcated from adjacent salivary glands. No evidence of infiltration or invasion was seen

## Discussion

The World Health Organization (WHO) defines BCA as a benign neoplasm consisting of organized basaloid cells with a prominent basal cell layer and basement membrane-like structure (2). It lacks myxochondroid stroma to differentiate it from pleomorphic adenoma. Three cellular subtypes are observed in this adenoma: solid, trabecular–tubular, and membranous. The typical clinical picture of basal cell adenoma is a firm, painless, slow-growing mass involving the parotid gland in middle-aged women (3). The involvement of other sites, such as the submandibular gland, sublingual gland, and minor salivary gland, is scarcely reported. 

BCA is a distinct type of salivary gland monomorphic tumour that closely mimics basal cell skin lesions. Solid BCA is produced by compactly arranged small cells, while the trabecular–tubular subtype is composed of narrow bands, ductal structures, or both. The membranous subtype consists of external cells arranged in a barrack pattern and an intense hyalinized basal membrane (4). Interestingly, our histological patterns were a combination of trabecular–tubular and solid types.

Computed tomography (CT) is a commonly employed radiological choice for most BCA cases, even though BCA characteristics on imaging are not well defined (5). However, we believe ultrasound is a valuable tool for preliminary scanning of this entity in the case of a superficial and accessible lesion, since it is more cost-effective. Ultrasound can easily reveal any evidence of calcification or necrosis required to distinguish it from malignancy before proceeding with CT. However, if the lesion is deeply seated, not palpable, or large enough to cause compressive symptoms, then CT is indicated.

Further, histological findings are essential to diagnose BCA, as the lesions mimic several benign and malignant salivary and non-salivary gland tumours, which are otherwise extremely challenging to determine on biopsy. Some authors have reported using FNAC for diagnosis, but the results were often inconclusive and even differed when repeating the procedure twice (6,7); therefore, the diagnosis could only be made after excisional biopsy (4,7). FNAC has a high sensitivity and specificity for non-neoplastic lesions but only intermediate sensitivity for malignant neoplasms, hence the possibility of misdiagnosis (8). 

The absence of mitoses, necrosis, any invasion into the surrounding tissue, or perineural invasion distinguishes BCA from its malignant counterparts, such as basal cell adenocarcinomas. Moreover, since there is only limited evidence supporting the use of immunohistochemistry markers for the differentiation of tumours (7), we recommend using a true-cut biopsy as the second line of investigation after an inconclusive FNAC to ascertain a definitive diagnosis preoperatively. 

Pleomorphic adenoma, adenoid cystic carcinoma, and basal cell adenocarcinoma are among the differentials for his presentation, with distinct prognostic effects and surgical procedures for each entity. Basal cell adenocarcinoma can be differentiated from BCA due to its abundant mitotic figures with infiltrative growth; 5% of the cells are also positive for K167 staining (4). 

In adenoid cystic adenocarcinoma, dark external cells and a pool of epithelial cells are arranged in a stack pattern with thick basal membrane–like structures (6). Therefore, ascertaining the exact diagnosis before embarking on surgery is of utmost importance. Most BCAs are manageable with only surgical excision or superficial gland removal, except for the membranous subtype, which needs complete gland resection. This is because of its high recurrence compared to the trabecular-tubular and solid variants, which have almost none. Also, although extremely rare, malignant transformation in the membranous type is more common than in other types (3).

## Conclusion

Basal cell adenoma is a particular type of salivary gland monomorphic tumour that strongly resembles the skin’s basal cell lesions. Although a few authors advocate the surgical excision of tumours to arrive at the final diagnosis, we strongly propose a definitive diagnosis preoperatively. This is attributable to its differential diagnosis, which may significantly impact the prognosis and the correct management of each diagnosis.
